# The space where aging acts: focus on the GABAergic synapse

**DOI:** 10.1111/acel.12605

**Published:** 2017-05-12

**Authors:** Aleksandra Rozycka, Monika Liguz‐Lecznar

**Affiliations:** ^1^ Department of Molecular and Cellular Neurobiology Nencki Institute of Experimental Biology Polish Academy of Sciences 3 Pasteur Street Warsaw 02‐093 Poland

**Keywords:** ageing, aging, GABA, GABAergic, postsynaptic, presynaptic, synapse

## Abstract

As it was established that aging is not associated with massive neuronal loss, as was believed in the mid‐20th Century, scientific interest has addressed the influence of aging on particular neuronal subpopulations and their synaptic contacts, which constitute the substrate for neural plasticity. Inhibitory neurons represent the most complex and diverse group of neurons, showing distinct molecular and physiological characteristics and possessing a compelling ability to control the physiology of neural circuits. This review focuses on the aging of GABAergic neurons and synapses. Understanding how aging affects synapses of particular neuronal subpopulations may help explain the heterogeneity of aging‐related effects. We reviewed the literature concerning the effects of aging on the numbers of GABAergic neurons and synapses as well as aging‐related alterations in their presynaptic and postsynaptic components. Finally, we discussed the influence of those changes on the plasticity of the GABAergic system, highlighting our results concerning aging in mouse somatosensory cortex and linking them to plasticity impairments and brain disorders. We posit that aging‐induced impairments of the GABAergic system lead to an inhibitory/excitatory imbalance, thereby decreasing neuron's ability to respond with plastic changes to environmental and cellular challenges, leaving the brain more vulnerable to cognitive decline and damage by synaptopathic diseases.

## Introduction

Aging is a physiological process that alters brain function, thereby resulting in behavioural changes, memory decline and cognitive impairments. Those changes depend on many factors and are related to structural, neurochemical and physiological processes in the brain (Burke & Barnes, 2006; Ouda *et al*., 2015). The age‐related cognitive impairments include numerous functions, such as learning, attention, working memory and executive functions (Burke & Barnes, 2006; Mattay *et al*., 2006).

Cognitive aging is a consequence of molecular and biochemical aging, which results in metabolic, hormonal and immune dysregulation, increased oxidative stress and inflammation, altered neurotransmission and reduced neurotrophic support of neural cells (Sibille, 2013). Alterations in gene expression, influencing the levels of proteins in many biological pathways, can be regarded as a hallmark of molecular aging. Changes in the biochemical composition of neural cells, which affect the efficiency of their synapses and whole circuits, impair the plasticity of the brain, that is the ability to reorganize, learn and remember. In this way, the disturbances of synaptic machinery profoundly contribute to the cognitive impairments as well as to the age‐related brain disorders.

The majority of studies concerning the plasticity of neural circuits have focused on excitatory synapses. However, the role of inhibitory interactions in neuroplastic changes has recently been widely recognized (Letzkus *et al*., 2011; Castillo *et al*., 2011; Kullmann *et al*., 2012). The most basic role of inhibitory neurons is to control the excitability of the principal cells, ensuring a proper homoeostatic balance and preventing runaway excitation (Karmarkar & Buonomano, 2006). In sensory systems, responses of cortical neurons are shaped by the temporal interplay between excitatory thalamocortical input and local cortical inhibition (Miller *et al*., 2010). Rapid reduction in excitation restricts the window that is available for temporal summation, enabling neurons to act as coincidence detectors and thus increasing temporal precision (Pouille & Scanziani, 2001). Interneurons are also involved in the phenomenon called the inhibitory sharpening of receptive fields (Foeller *et al*., 2005; Froemke *et al*., 2007; Carvalho & Buonomano, 2009). Strong network inhibition suppresses the excitatory population response to tonic input, providing the circuit with an intrinsic mechanism enabling precise contrast‐gain control. Therefore, even though excitatory neurons are a large majority of cortical neurons, local inhibitory interneurons shape their firing and timing. There is increasing support for the hypothesis that disruption of inhibitory circuits is responsible for some of the clinical features of many neuropsychiatric and neurodegenerative disorders, such as schizophrenia, autism, depression, epilepsy, Alzheimer's disease and Parkinson's disease. Many of them have been proposed to be synaptopathies – diseases related to the dysfunction of synapses (Brose *et al*., 2010). Brain aging is, in this context, considered a phenomenon promoting biological alterations associated with the above‐mentioned disorders, resulting in so‐called late‐onset diseases.

The difficulty in understanding the mechanisms of interneurons aging, along with its relationship to plasticity impairments, cognitive decline and brain disorders, lies in the tremendous diversity of inhibitory neurons. Inhibition can be performed by perisomatically, dendritically or axonally targeting interneurons, which can be devoted to different inhibitory tasks (Royer *et al*., 2012). Interneurons differ in their connectivity, input/output efficacy, membrane properties and firing pattern, and all those features determine their postsynaptic impact on target cells (Gupta *et al*., 2000; Beierlein *et al*., 2003; Markram *et al*., 2004; Tremblay *et al*., 2016). Furthermore, over 20 subtypes of neurons using GABA as a neurotransmitter have been recognized based on their anatomical, physiological and molecular features, such as the presence of characteristic additional markers like other neurotransmitters, cell surface markers, transcription factors, neuropeptides and calcium‐binding proteins (Ascoli *et al*., 2008).

Nevertheless, this diversity makes interneurons a potent and complex regulatory machinery controlling the physiology of neural circuits (Maffei, 2011), and their molecular and biochemical aging can significantly contribute to the cognitive deficits observed in the aged brain. The role of neuroplasticity is to compensate for those age‐related changes and to maintain the proper function of inhibitory circuits, supporting the balance between excitation and inhibition and the correct cognitive performance.

In this review, we collected information regarding age‐related alterations in GABAergic neurons and synapses, trying to relate them to the impairments of plasticity that are observed in old age.

## Numbers of GABAergic neurons and synapses

Originally, it was postulated that inhibitory deficits observed in aging and neurological disorders are due to decreases in the number of interneurons, and many studies have supported this hypothesis. In aging, Hua *et al*. (2008) observed that although the total density of neurons remained unaltered in visual cortex of cats, the density of GABA‐immunoreactive (GABA‐IR) neurons was significantly lower. A loss of GABAergic interneurons was shown in the aged rat hippocampus together with reduced inhibition of dendritic input from the entorhinal cortex (Stanley *et al*., 2012). Other studies have shown loss of inhibitory synaptic contacts. Gradual loss of symmetric synapses was confirmed, for example in layer 2 and 3 of the monkey prefrontal cortex by Peters *et al*. (2008), and decreased density of GABAergic boutons was found in the frontal and parietal cortices of aged rats (Majdi *et al*., 2007). Declines in the number and complexity of GABAergic terminals unrelated to neuronal loss were observed in the hippocampus of a mouse model of Alzheimer's disease (Rubio *et al*., 2012; Soler *et al*., 2017). Recent studies suggest that inhibitory deficits may also be related to the dysfunction of particular interneuronal subpopulations and their circuits (Marín, 2012). In accordance with this hypothesis, Akbarian *et al*. (1995) demonstrated reduced expression of GAD in the absence of significant cell loss in schizophrenic brains. However, dysfunction and pruning of synaptic endings may occur simultaneously, preceding neuronal loss as observed in Alzheimer disease (Shankar & Walsh, 2009).

To conclude, age‐related regression, loss and dysfunction of inhibitory synapses can be observed in many brain structures (Dickstein *et al*., 2007; Morrison & Baxter, 2012).

### Parvalbumin (PV)‐containing interneurons

Fast‐spiking PV interneurons are the most numerous GABAergic cells, and they not only execute feedforward and feedback inhibition but also are responsible for generating gamma‐frequency oscillations. In aging, loss of PV‐containing neurons has been shown in the somatosensory, auditory and motor cortices of rats (Miettinen *et al*., 1993; Ouda *et al*., 2008) as well as in the hippocampus. On the other hand, an unchanged number of PV cells was detected in the tissue of aged healthy human cerebral cortex and hippocampus (Bu *et al*., 2003). Nevertheless, decreases in the number of PV neurons and their dysfunction have been associated with the loss of gamma oscillations in schizophrenic patients who manifested deficits in working memory and executive functions (Torrey *et al*., 2005; Sohal *et al*., 2009). The link between altered function of PV neurons and neurological/psychiatric disorders has also been confirmed for epilepsy, autism, Alzheimer's disease and depression (Rossignol, 2011).

### Somatostatin (SOM)‐containing interneurons

The number of SOM neurons has been reported to be decreased in the somatosensory and motor cortices of aged rats (Miettinen *et al*., 1993), as well as in the human auditory cortex beginning in midlife (Ouellet & de Villers‐Sidani, 2014). Selective loss of SOM cells has also been shown in rat hippocampus (Stanley *et al*., 2012). Moreover, lower levels of mRNA for SOM have been observed in the frontal, temporal, motor, visual, and somatosensory cortices and hippocampus of primates (Hayashi *et al*., 1997).

Many pathological conditions are associated with alterations in SOM interneurons (Lin & Sibille, 2013). These cells are vulnerable to seizure‐induced death, and a decrease in their number is considered a hallmark of epileptic hippocampus (Clynen *et al*., 2014). Alterations in the somatostatinergic system have been reported in depression, schizophrenia, bipolar disorder and Alzheimer's disease (see Liguz‐Lecznar *et al*., 2016). Saito *et al*. (2005) postulated that a decrease in SOM expression can act as a trigger for amyloid β accumulation, thereby contributing to late‐onset Alzheimer's disease.

### Calbindin (CB)‐ and Calretinin (CCR)‐containing interneurons

Bu *et al*. (2003) reported an age‐related decline in CB‐immunopositive cell density in the human visual cortex and parahippocampal gyrus and a decline in CCR neuronal density in the auditory cortex (see also Ouda et al. 2012). Loss of CB‐positive interneurons was reported by Potier *et al*. (2006) in the aged rat hippocampus together with lower GABAergic inhibition.

Alterations in CB‐positive interneurons were detected in a mouse model of autism (Rossignol, 2011). Coincident age‐related loss of CB neurons and tangle formation in the basal forebrain in Alzheimer's disease were shown by Ahmadian *et al*. (2015).

### Other GABAergic subpopulations

Roles of vasointestinal peptide (VIP)‐containing interneurons have been confirmed in the pathology and treatment of such neurological disorders as Alzheimer's disease, Parkinson's disease and autism spectrum disorders (White *et al*., 2010). Decreased GABAergic inhibition due to deficits in forebrain neuropeptide Y (NPY)‐containing, SOM‐containing and PV‐containing neurons has been proposed as a possible pathogenic mechanism of autism spectrum disorder (Sgadò *et al*., 2013). Further, NPY neurons have been shown to degenerate selectively in a mouse model of amyloidosis and tauopathy (Loreth *et al*., 2012).

In aging, Cha *et al*. (1997) observed a substantial loss of VIP interneurons in sensory cortex of aged rats. An age‐related loss of NPY neurons was observed in the rat retrosplenial, frontal, occipital and temporal cortical areas, as well as in the hippocampus (Cha *et al*., 1997). Additionally, a decline in NPY cells has been reported in the auditory cortex of rats (Ouellet & de Villers‐Sidani, 2014).

Notably, the impact of aging on the expression of distinct GABAergic markers can be not only species‐specific but also strain‐specific, and such differences have been reported for parvalbumin in the rat strains Long–Evans vs. Fischer 344 (Ouda *et al*., 2008) as well as for CB and CCR in mice when comparing the strains CBA/CaJ and C57BL/6J (Zettel *et al*., 1997).

Together with decreased neurotransmitter release and reduced responsiveness of postsynaptic neurons, the depletion of GABAergic neurons and their synaptic contacts can result in loss of the excitatory/inhibitory balance. This prompts plasticity impairment, and if compensatory mechanisms are ineffective, such imbalance can generate cognitive impairments or even trigger pathophysiological pathways leading to disease.

## Functional changes

The dysregulation of GABAergic signalling in aging is a widely accepted phenomenon. However, there is no single universal scheme for age‐related alterations of intrinsic neuronal properties, and the direction of changes depends on the structure and neuronal population. In contrast to the prefrontal cortex, for which experimental data support increased inhibition with age (Luebke *et al*., 2004; Bories *et al*., 2013; Bañuelos *et al*., 2014), there is strong evidence for decreased intracortical inhibition in sensory systems and the hippocampus. Increased spontaneous activity and a decreased signal‐to‐noise ratio were observed in the visual system of aged cats and rats (Wang *et al*., 2006; Lehmann *et al*., 2012). Using a paired‐pulse stimulation paradigm, Schmidt *et al*. (2010) confirmed an age‐dependent reduction in functional inhibition in the parietal cortex, and David‐Jürgens & Dinse (2010) confirmed this in rat somatosensory cortex. Furthermore, the same phenomenon has been observed in the human somatosensory system (Cheng & Lin, 2013), where it was associated with impaired tactile acuity (Lenz *et al*., 2012), as well as in primary motor cortex, where weaker resting‐state inhibition was associated with poorer manual motor performance (Heise *et al*., 2013). Several studies using the whole‐cell patch‐clamp method have demonstrated a reduced frequency of spontaneous inhibitory postsynaptic potentials (IPSPs), as well as reduced amplitude and frequency of GABA receptor (GABAR)‐mediated currents, in the aged hippocampus (Potier *et al*., 2006; McQuail *et al*., 2015). This altered balance of excitatory and inhibitory pathways in local brain circuits influences the neuroplastic potential of the aging nervous system.

## Aged GABAergic synapses and plasticity

There is ample evidence that GABAergic neurons and synapses undergo plastic changes. The significance of GABAergic synapse plasticity is highlighted by the number of experiments on this topic (Gubellini *et al*., 2001; Kawaguchi & Hirano, 2002; Patenaude *et al*., 2003; Lien *et al*., 2006; Maffei *et al*., 2006). Furthermore, it has been shown that the learning process is associated with modification of inhibitory GABAergic neurotransmission and reorganization of cortical activation (Froemke *et al*., 2007; Tokarski *et al*., 2007; Brosh & Barkai, 2009; Jasinska *et al*., 2010; Urban‐Ciecko *et al*., 2010).

Animal studies investigating the impact of aging on the GABAergic system have mainly demonstrated reduced inhibition with age, but in some specific areas (such as the prefrontal cortex), inhibition has been reported to increase with age (Potier *et al*., 2006;  Stanley *et al*., 2012; Bories *et al*., 2013). Regardless of whether inhibition in the particular brain area was attenuated or augmented, changes in specific inhibitory interneurons functioning have been found to contribute to cognitive, memory and behavioural changes (Stanley *et al*., 2012; Bories *et al*., 2013).

Aging is associated with peripheral deafferentation, resulting in compensatory mechanisms in the appropriate CNS pathways. This phenomenon, described as adaptive plasticity, can be found in different sensory systems and leads to a selective downregulation of inhibition (Caspary *et al*., 2008), which is considered a type of ‘negative plasticity’ (Mahncke *et al*., 2006). Consequences of reduced inhibition include impaired tactile acuity and diminished visual and auditory signal‐to‐noise coding (Leventhal *et al*., 2003; Yu *et al*., 2006; Hua *et al*., 2008; David‐Jürgens & Dinse, 2010; Kamal *et al*., 2013). Moreover, an association between weaker inhibition and lower manual motor performance was confirmed in the human motor system (Heise *et al*., 2013). All the above‐mentioned changes are indications of homoeostatic plasticity that serves to re‐establish the balance between the excitatory and inhibitory systems.

**Figure 1:**
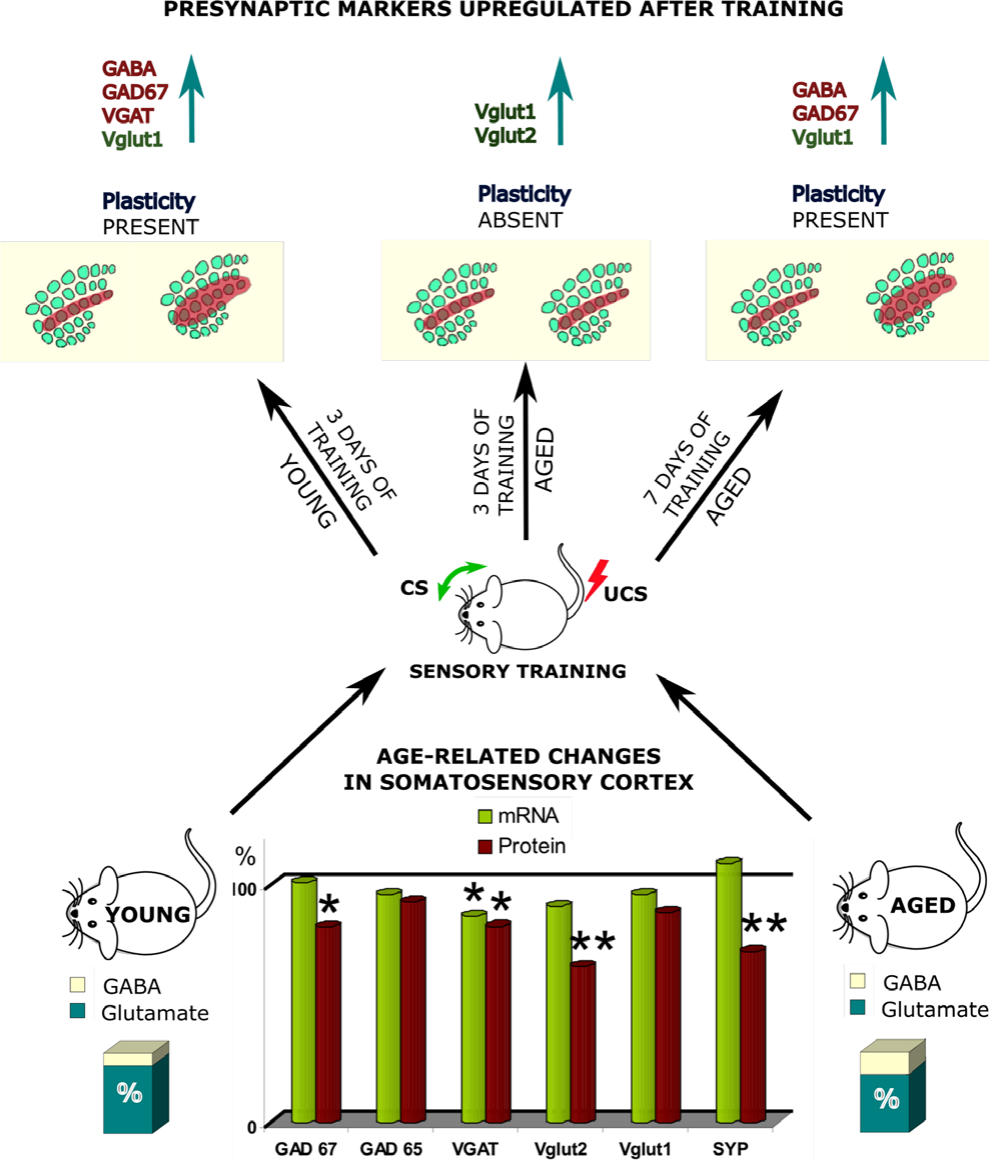
Age‐related changes influence learning‐dependent plasticity in the mouse somatosensory cortex. From the bottom: Aging alters the level of several proteins related to glutamatergic and GABAergic neurotransmission in the somatosensory (SI) cortex of mice. mRNA for those proteins, with the exception of VGAT, is unaltered. Due to the decreased level of glutamate with an unchanged level of GABA in aged mouse SI, a lower glutamate‐to‐GABA ratio can be observed, suggesting an imbalance between excitation and inhibition. When submitted to short (3‐day) sensory training based on a classical conditioning paradigm (conditioned tactile stimulus paired with an unconditioned aversive stimulus), young mice elaborate functional cortical plasticity. This plastic change is visible, after mapping brain activity using the 2‐DG method, as an enlargement of the functional cortical representation of the trained row of vibrissae. Aged (1‐year‐old) mice do not demonstrate such plasticity after short training, even though they present the conditioned response similarly to the young mice. After longer (7‐day) conditioning, aged animals also elaborate the plastic change in SI. Training‐induced plasticity is associated with increased levels of GAD67 and GABA in SI. This may serve to attenuate the increased activity suggested by upregulation of Vglut1 and Vglut2 after training. In young animals, training also upregulates VGAT, which can further support GABAergic neurotransmission. In aged animals, probably due to a significant reduction in the VGAT mRNA level, the upregulation of VGAT expression after training is impossible, and that decreases the ability of the GABAergic system to effectively respond to increased demands on the inhibitory drive. Thus, aged mice require more time to elaborate the plastic change. GAD65 and GAD67 – glutamic acid decarboxylases; SYP – synaptophysin; VGAT – vesicular GABA transporter; Vglut1 and Vglut2 – vesicular glutamate transporters; CS – conditioned stimulus; UCS – unconditioned stimulus, 100% – expression level in young animals.

The situation becomes even more complicated when an upregulation of inhibitory system is necessary to induce plasticity or to accomplish some cognitive task (Scelfo *et al*., 2008; Petrini *et al*., 2014; Wang & Maffei, 2014). Plasticity of cortical maps induced by sensory training based on classical conditioning paradigms is a type of plasticity that requires upregulation of the GABAergic system (Siucinska, 2006; Tokarski *et al*., 2007; Jasinska *et al*., 2010; Liguz‐Lecznar *et al*., 2011; Posluszny *et al*., 2015). Following the training in which stroking a row of whiskers is paired with a tail shock for three consecutive daily sessions, functional cortical representation of stimulated whiskers expands in young animals (Siucinska, 2006) (Fig. 1). This plastic change is accompanied by an increase in the number of SOM neurons in the representation of the trained row (Cybulska‐Klosowicz *et al*., 2013), increased inhibitory synaptogenesis, increased tonic inhibition of fast‐spiking neurons and increased IPSC frequency. We found that in aged mouse somatosensory cortex, the same training is ineffective, and learning‐induced plasticity of cortical whisker representations could not be observed (Liguz‐Lecznar *et al*., 2015), In young mice, the training causes an increase in the GABA content in the barrel cortex, as measured by HPLC, while in aged mice, no such effect was found (Liguz‐Lecznar *et al*., 2015). However, the plastic change could be induced with a longer training paradigm (Fig. 1), and then, the increase in GABA level was observed. These data led us to postulate that the decrement in plasticity observed in aged animals was not due to neurodegeneration but rather due to an ineffectiveness of mechanisms governing plasticity: specifically, the upregulation of the GABAergic system in response to increased demands on the inhibitory drive (Liguz‐Lecznar *et al*., 2015) (Fig. 1).

In aging, two plasticity‐related phenomena interfere with each other: adaptive changes and experience‐dependent plasticity. Some of these adaptive mechanisms may also be shared with degenerative cascades, and once activated, they may lead to cognitive impairment and disease (Cotman & Anderson, 2000). Thus, the strong plastic potential and involvement of experience‐dependent plasticity may allow the brain to overcome the negative effects of aging. This was confirmed in numerous experiments that demonstrated the beneficial result of a stimulating environment and physical/cognitive training on cognitive functions such as memory (Sale *et al*., 2014; Consortium T.t.B., 2017) and also based on the effects of enriched environment on Aβ accumulation during aging (Li *et al*., 2013), hippocampal neurogenesis and the level of neurotransmitters (Segovia *et al*., 2006).

## Synapse – the space where aging acts

The structure of the synapse is complex, and maintaining the correct composition of presynaptic and postsynaptic terminals as well as precise coordination of presynaptic and postsynaptic activities is essential for cognitive functions (including learning and memory consolidation) and for synaptic plasticity (Abraham, 2008). In fact, synaptic dysfunction has been documented to be an early event in the course of many neurodegenerative diseases such as Huntington's disease (Li *et al*., 2001), temporal lobe epilepsy (Ratté & Lacaille, 2006) and Alzheimer's disease (Masliah *et al*., 2006). In physiological aging, the decline in synaptic density, pruning of the dendritic tree, loss of dendritic spines, structural changes within the presynaptic active zone and alteration of receptors for different neurotransmitters have been detected across the nervous system (Burke & Barnes, 2006). To maintain stable neurotransmission and enable optimal synaptic performance, synapses are under the control of homoeostatic mechanisms that act through compensatory adjustments in synaptic strength and cellular excitability (Turrigiano *et al*., 1998; Burrone *et al*., 2002). Aging‐related changes can be detected in excitatory and inhibitory synapses and often lead to an imbalance of excitation and inhibition, which can cause further instability in neuronal networks and gating defects related to cognitive impairments (Marín, 2012).

Many recent genomewide human and animal studies show that the proportion of genes undergoing an age‐dependent regulation is relatively small, representing no more than 5% of the entire genome. However, because research by Loerch *et al*. (2008) using species‐specific genome‐scale microarrays revealed that genes associated with GABAergic inhibitory function are significantly more strongly age‐downregulated in humans than in rhesus monkeys or mice, results regarding gene alterations with aging should be interpreted cautiously.

The postulated pattern is that neuron‐related transcripts involved in signalling and cellular communication are downregulated, while the opposite can be observed for glial‐related genes engaged in inflammation and cellular defence (see Sibille, 2013). Despite the relatively stable transcriptome, the brain shows many age‐related protein alterations, which indicates mRNA–protein decoupling (Wei *et al*., 2015). Our analysis of aging‐related changes in the expression of several presynaptic markers also revealed that they were downregulated with age, mainly at the protein level (Liguz‐Lecznar *et al*., 2015) (Fig. 1).

## Inside the aged GABAergic synapse

### Presynaptic impact

Existing data support the idea that synaptic dysfunction resulting from altered neurotransmission is a significant contributor to aging‐related impairments of nervous system functioning (Morrison & Baxter, 2012). Several conditions must be met to maintain synaptic functionality and unaltered neurotransmission: preservation of proper synaptic structure, coordination of synaptic vesicle release and membrane excitability, and effective integration of retrograde signals from the postsynaptic terminal (Azpurua & Eaton, 2015).

Presynaptic terminals serve to release neurotransmitters and contain an extensive array of proteins that are expressed in precise quantities, located in specific areas and designated for particular functions (Garner *et al*., 2000; Fritschy *et al*., 2012). Together, they constitute a dedicated system to ensure accurate, efficient and reliable synaptic transmission.

Consistent with age‐related decreases in inhibition, many studies have demonstrated decreases in the synthesis and level of GABA. GABA is synthesized from glutamate by its decarboxylation, catalysed by the two isoforms of the enzyme glutamate decarboxylase (GAD65 and GAD67) (Erlander *et al*., 1991). Many studies have reported aging‐associated alterations in the levels of both GAD isoforms in different brain areas. In most cases, the number of GAD‐IR neurons, the amount of protein or the mRNA level decreased with age (Gold & Bajo, 2014). Ling *et al*. (2005) reported reduced levels of GAD65 and GAD67 mRNAs, and Burianova *et al*. (2009) reported an age‐related decrease in the protein levels of GAD65 and GAD67 in the rat auditory cortex. They suggested that the observed changes may contribute significantly to the deterioration of hearing function. In the aging mouse barrel cortex on the other hand, age‐related decreases in the protein levels of both GAD isoforms were observed without significant changes in mRNA expression (Liguz‐Lecznar *et al*., 2015). Loss of GAD level in interneurons was also observed in aging visual cortex and the hippocampus (Shi *et al*., 2004; Stanley & Shetty, 2004; Liao *et al*., 2016). Such results are not always accompanied by alterations in the GABA levels in aging tissue. In our experiments, despite decreased levels of GAD65 and 67 in aging mice, we did not detect a significant decrement in the GABA level in the somatosensory cortex (Liguz‐Lecznar *et al*., 2015). Nevertheless, although GAD expression may not directly translate to basic GABA level, its depletion may play a restrictive role during the induction of plastic changes.

### GABA transport

After release from presynaptic terminals, GABA is quickly removed from the extracellular space by a system of transporters, which are transmembrane proteins with four distinct subclasses: GAT‐1, GAT‐2, GAT‐3 and BGT‐1. They regulate the extracellular concentration of GABA and prevent excessive activation of GABARs (Scimemi, 2014). However, before it can be released, GABA must first be packed into synaptic vesicles. This step is mediated by the vesicular GABA transporter (VGAT) (Chaudhry *et al*., 1998).

Information about the role of GATs and changes in their expression related to brain aging is sparse. A few studies reported a significant reduction in GAT‐1 expression in specific brain areas, such as the rat medial prefrontal cortex (Bañuelos *et al*., 2014) and the human frontal cortex (Sundman‐Eriksson & Allard, 2006). Similarly, little is known about how VGAT expression or activity changes during aging. A study by Canas *et al*. (2009) presented an age‐related continuous decrease in the VGAT level in the rat hippocampus. Additionally, our experiments on the barrel cortex of aged mice demonstrated decreased VGAT mRNA and protein levels (Liguz‐Lecznar *et al*., 2015) (Fig. 1).

## Postsynaptic impact

### GABA receptors

The main and potentially most important postsynaptic components that may influence the action of inhibitory synapses are GABA receptors (GABARs). They are divided into two classes: fast‐acting ionotropic GABA_A_ and slower acting metabotropic GABA_B_ receptors (GABA_A_Rs and GABA_B_Rs, respectively) (Pinto *et al*., 2010).

### GABA_A_Rs

GABA_A_Rs are ligand‐gated chloride‐ion channels that regulate fast inhibitory neurotransmission in the brain (Bowery *et al*., 2002). Biophysical properties of the receptor are determined by its subunit composition (Koksma *et al*., 2005), which can be regionally specific in the brain (Sieghart & Sperk, 2002). The number of postsynaptic GABA_A_Rs plays a crucial role in shaping synaptic plasticity because it is proportional to the functional strength of GABAergic synapses (Nusser *et al*., 1997; Luscher *et al*., 2011). Thus, even a slight decrease in the number of GABA_A_Rs (by 5–35%) can result in noticeable behavioural changes (Shen *et al*., 2010; Luscher *et al*., 2011). It has been shown that age‐related changes in the composition of GABA_A_R subunits that affect channel kinetics, ligand binding and ion specificity contribute to cognitive impairments (Rissman & Mobley, 2011). In rat auditory cortex, Schmidt *et al*. (2010) showed a decline in the α5 subunit of GABA_A_R, and in the auditory thalamus, Richardson *et al*. (2013) demonstrated reduced activation and expression of high‐affinity GABA_A_Rs, mediating tonic inhibition. Caspary *et al*. (2013) showed that in the aged rat auditory cortex, some subunits of GABA_A_R were downregulated (α1, β_1_, β_2_, γ_1_ and γ_2_ subunits) while the α3 subunit was upregulated, and this effect was specific for particular cortical layers. Yu *et al*. (2006) revealed changes in the regional expression of GABA_A_R subunits, with decreases in the α3 and α5 subunits in rat motor and somatosensory cortex, a transient decrease in the γ2 subunit and no alterations in the expression levels of α1 and α2. Gutiérrez *et al*. (1997) showed age‐related changes in the protein and mRNA levels of the α1 and γ2 subunits in the rat cortex. In the hippocampus, an age‐related upregulation of the α1 subunit of GABA_A_R was reported in rats, while a decrease was observed in monkeys (Gutiérrez *et al*., 1996; Rissman *et al*., 2007). Moreover, the γ2 subunit was downregulated in the aged rat hippocampus (Yu *et al*., 2006).

Those results suggest that aging, through quite subtle changes in the subunit composition of GABA_A_R, can change its physiology and alter inhibitory synaptic transmission.

### GABA_B_Rs

GABA_B_Rs constitute a group of heterodimeric G protein‐coupled receptors. Those transmembrane proteins mediate slow inhibitory responses and can be expressed on the presynaptic or postsynaptic side. Presynaptically localized (on GABAergic and glutamatergic terminals – autoreceptors) GABA_B_R regulate neurotransmitter release, while receptors localized postsynaptically or extrasynaptically on pyramidal neurons mediate tonic inhibition (Wang *et al*., 2010). Depending on the brain region, aging induced different alterations in GABA_B_Rs, thereby causing different changes in behaviour and learning. Binding of GABA_B_Rs was reduced in the inferior colliculus and cortex of aged rats (Milbrandt *et al*., 1994; Turgeon & Albin, 1994; Caspary *et al*., 1995). In the hippocampus, a selective loss of the GABA_B_R1 subunit was observed in aged rats with a spatial learning impairment (McQuail *et al*., 2012). On the other hand, Bañuelos *et al*. (2014) reported that GABA_B_R expression was negatively correlated with working memory performance. It was further shown that administration of a GABA_B_R antagonist enhanced working memory in aged rats and ameliorated deficits in olfactory discrimination learning (Lasarge *et al*., 2009). However, the possible effects of age‐related GABA_B_R alterations can be complex, because its presynaptic action inhibits neurotransmitter release and thus contributes to a net effect of enhancing excitatory signalling, while its postsynaptic activation induces postsynaptic inhibitory currents and activates CREB2, a transcription factor involved in the regulation of memory formation suppressor genes (Helm *et al*., 2005; Emson, 2007).

Although GABARs have a substantial impact on synaptic plasticity by controlling LTP, synaptic strength and maturation of dendritic spines, many other components of the inhibitory postsynaptic density (PSD) in GABAergic synapses contribute to controlling the function of postsynaptic terminals.

### Gephyrin and other proteins of the inhibitory PSD

Gephyrin, initially characterized as a tubulin‐binding protein, is described as a neuronal assembly protein that anchors other proteins and is the major inhibitory scaffolding component. It is a membrane‐associated protein that co‐localizes with both glycine and GABA receptors (Wang *et al*., 1999; Craig & Boudin, 2001), and its level can be used as an indicator of the total amount of GABA_A_R (Pinto *et al*., 2010). Gephyrin functioning is regulated by phosphorylation, and gephyrin can be added or removed from the postsynaptic scaffold (Fritschy *et al*., 2012). Clustering and functioning of gephyrin is regulated by interactions with many other proteins, including neuroligin‐2 (NL2), which is a postsynaptic adhesion molecule (Maćkowiak *et al*., 2014), and collybistin, which regulates the aggregation of gephyrin and GABA_A_R (Poulopoulos *et al*., 2009). Little is known about the age‐related changes in those proteins. A decrease in gephyrin expression with age was observed in the human visual cortex (Pinto *et al*., 2010). On the other hand, an increase in gephyrin expression was detected in the parietal cortex of cognitively impaired aged rats (Majdi *et al*., 2009).

## Similarities between age‐related and pathology‐related changes within GABAergic synapses

Alterations similar to those seen in aging can be observed in neurological and psychiatric diseases. In schizophrenia, GAD67 mRNA expression in prefrontal cortex is downregulated in a subset of parvalbumin‐containing interneurons (Volk & Lewis, 2005). Reduced levels of GAD65 and 67 can be observed in parietal and cerebellar cortices in autism (Fatemi *et al*., 2002). A diminished number of GABA_A_Rs was confirmed in the cell membranes of Alzheimer's disease brains (Bernareggi *et al*., 2007). Analysis of mRNA expression and the proportions of GABA receptor subunits revealed downregulation of the α1 and γ2 subunits with concomitant upregulation of α2, β1 and γ1 transcripts in AD brains (Limon *et al*., 2012).

Supporting this similarity, the reinforcement of the GABAergic system is regarded as a therapeutic strategy in age‐related cognitive decline (McQuail *et al*., 2015) as well as in several neurodegenerative diseases and neurological disorders (Stan & Lewis, 2012). Leventhal *et al*. (2003) have shown that stimulation of the GABAergic system with the GABA_A_R agonist muscimol resulted in improved properties of neurons in aged monkey visual cortex. In schizophrenia, a clinical trial with a benzodiazepine‐like drug specific for particular subtype of GABA_A_R revealed its effectiveness in memory improvement (Lewis *et al*., 2008). The therapeutic potential of GABA_B_R ligands has also been suggested for depression, epilepsy and Alzheimer's disease (see Kumar *et al*., 2013). In addition to the benefits of drugs attenuating amyloid‐induced synaptic dysfunction, several studies have demonstrated protection against Aβ‐induced neurotoxicity with a selective GABA_A_ receptor agonist (i.e. muscimol) in retinal, hippocampal and cortical neurons in rodents (see Nava‐Mesa *et al*., 2014). Additionally, more recent studies in preclinical models show that GABAergic cell grafting can be beneficial for treating schizophrenia, neuropathic pain, Alzheimer's disease and Parkinson's disease (Chohan & Moore, 2016; Shetty & Bates, 2016). Interestingly, such interneuron‐based transplantation (PV and SST neurons) was also demonstrated to be sufficient to induce cortical plasticity (Tang *et al*., 2014).

The connection between brain aging and late‐life or late‐onset diseases can be explained by the age‐by‐disease biological interaction model. According to this model, the common direction of many genes alterations in aging and diseases suggests that the ‘brain progressively moves with advancing age towards a state that is biologically more consistent with those observed in the context of neuropsychiatric and neurological disorders’ (Sibille, 2013). To this hypothesis, we add the role of synaptic plasticity, which, if sufficient, can support the neural circuits in resisting the detrimental effect of aging.

## Compensatory mechanisms in aging synapses

Our experiments revealed that despite the impairment of learning‐induced plasticity in aged animals, it was still possible to evoke but required longer training. Moreover, in the somatosensory cortex of those animals, we observed a distinct pattern of proteins with changed level after training, different from the pattern found in young animals (Liguz‐Lecznar *et al*., 2015) (Fig. 1). This suggests that young and aged animals can engage different mechanisms to accomplish the same process and that even aged synapses with some protein deficits can support plasticity (Fig. 1). This can most probably be achieved by some compensatory or adaptive changes in the synapse, and such changes have been seen in many aged brain regions. For example, it was suggested that the loss of inhibitory synapses in the aged prefrontal cortex may be offset by the upregulation of GABA synthesis (Luebke *et al*., 2004; Bories *et al*., 2013). In the hippocampus, an age‐related increase in synaptophysin expression has been suggested to compensate for deficits in spatial learning and memory (Benice *et al*., 2006; Kumar & Thakur, 2015). Experimental data confirmed that a loss of GABAergic input in the hippocampus during aging can be compensated for at the postsynaptic level by upregulation of GABA_A_R expression with higher sensitivity to GABA (Ruano *et al*., 2000; Vela *et al*., 2003).

Such compensational changes, if sufficient, would help to preserve, to some extent, plastic potential and cognitive flexibility. Moreover, de Villers‐Sidani *et al*. (2010) have shown, in the rat auditory cortex, that age‐related auditory processing deficits (i.e. temporal coding and cortical desynchronization) and structural changes in parvalbumin‐containing interneurons can be largely reversed with behavioural training. This demonstrates that sensory experience strongly influences the cognitive capabilities in aging and has the potential to reverse the alterations in inhibitory interneuron morphology and cognitive decline in the aged brain.

## Conclusions

Age‐related loss of synaptic contacts, decreased neurotransmitter release and reduced postsynaptic responsiveness to neurotransmitters result in a decline in synaptic strength, contributing to age‐related cognitive decline. Molecular aging, defined as age‐related transcriptome changes, and biochemical protein‐related alterations within synapses weaken the plastic potential of neurons. Inhibitory neurons, despite being in the minority, are powerful regulators of neuronal excitability and, being particularly susceptible to aging‐related alterations, are involved in many aging‐induced cognitive impairments and brain disorders.

In the model of aged mouse somatosensory cortex, we have shown that although potential for learning‐related plasticity is preserved there, the corresponding mechanisms are weakened and need longer stimulation to trigger plastic changes. We have postulated that the decreased effectiveness of the GABAergic system in the aged mouse somatosensory cortex contributes to the deficits in learning‐induced plasticity.

Based on the age‐by‐disease molecular interaction model, stating that aging of the brain overlaps with biological pathways implicated in multiple brain disorders, we suggest that biochemical alterations in synapses, leading GABAergic neurons to be unable to respond quickly to the increased demand on the inhibitory drive, are a cause of age‐related learning‐induced plasticity impairment, which is an intermediate stage of the transition from healthy aging to age‐related cognitive decline and then to disease (See Box 1). Pharmacological and/or environmental reinforcement of the GABAergic system seems to be a promising therapeutic target for aging‐related brain disorders.

Box 1 **Progressive age‐related alterations in inhibitory neurons resulting in a decline in synaptic plasticity and compensatory mechanisms are determining factors for the transition between healthy and pathological aging.**

Molecular aging (transcriptome alterations) and biochemical aging (protein alterations) result in synaptic alterations and deficits of inhibition, which generate an imbalance between excitation and inhibition. This results in deregulation of excitatory cell input/output, local neuronal circuit processing and induction of negative adaptive changes; together with decreased plasticity, these changes cause further dysfunction of synapses and finally synaptic loss. Weakened compensatory mechanisms are unable to cope with those alterations, and pathological neurochemical and neurophysiological processes are triggered, directing neurons towards disease.

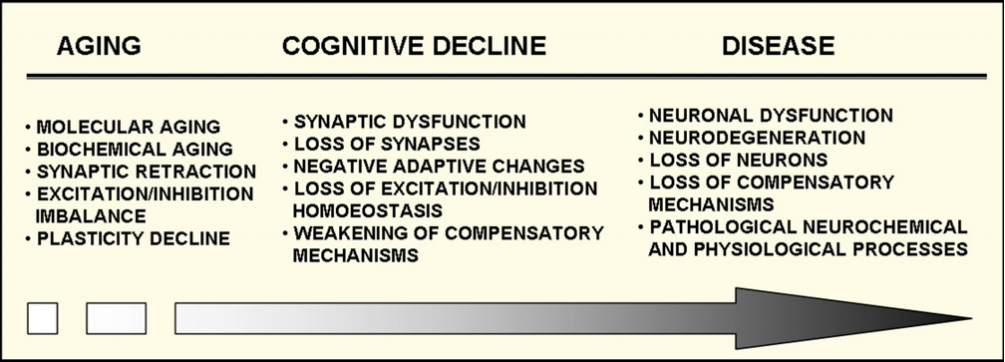

The consequences of age‐induced alterations in the synapse are difficult to interpret and predict. They are often specific for a given brain region or even a particular cell type, and the knowledge concerning the impact of aging on particular components of the synapse is fragmentary. While the extensive array of proteins present in presynaptic and postsynaptic terminals provides the possibility of functional compensation in age‐related deficits, it would be of particular importance to discover synaptic components and mechanisms that are altered in aging. Such knowledge would enable old synapses to be supported, thus maintaining proper neuronal signalling and counteracting the detrimental effects of aging.

## Author contributions

Both authors contributed equally to the data collection and to writing the manuscript.

## Funding

Work leading to this review was supported in part by a National Science Centre Grant (2013/09/B/NZ3/00540) and by Statutable Funds of the Nencki Institute of Experimental Biology PAS.

## Conflict of interest

None declared.
